# Multiple syntrophic interactions drive biohythane production from waste sludge in microbial electrolysis cells

**DOI:** 10.1186/s13068-016-0579-x

**Published:** 2016-08-02

**Authors:** Qian Liu, Zhiyong Jason Ren, Cong Huang, Bingfeng Liu, Nanqi Ren, Defeng Xing

**Affiliations:** 1 State Key Laboratory of Urban Water Resource and Environment, School of Municipal and Environmental Engineering, Harbin Institute of Technology, P.O. Box 2650, 73 Huanghe Road, Nangang District, Harbin, 150090 Heilongjiang China; 2 Department of Civil, Environmental, and Architectural Engineering, University of Colorado Boulder, Boulder, CO 80309 USA

**Keywords:** Biohythane, Waste sludge, Microbial electrolysis cell, Methane, Syntrophy, Microbial communities

## Abstract

**Background:**

Biohythane is a new and high-value transportation fuel present as a mixture of biomethane and biohydrogen. It has been produced from different organic matters using anaerobic digestion. Bioenergy can be recovered from waste activated sludge through methane production during anaerobic digestion, but energy yield is often insufficient to sludge disposal. Microbial electrolysis cell (MEC) is also a promising approach for bioenergy recovery and waste sludge disposal as higher energy efficiency and biogas production. The systematic understanding of microbial interactions and biohythane production in MEC is still limited. Here, we report biohythane production from waste sludge in biocathode microbial electrolysis cells and reveal syntrophic interactions in microbial communities based on high-throughput sequencing and quantitative PCR targeting 16S rRNA gene.

**Results:**

The alkali-pretreated sludge fed MECs (AS-MEC) showed the highest biohythane production rate of 0.148 L·L^−1^-reactor·day^−1^, which is 40 and 80 % higher than raw sludge fed MECs (RS-MEC) and anaerobic digestion (open circuit MEC, RS-OCMEC). Current density, metabolite profiles, and hydrogen-methane ratio results all confirm that alkali-pretreatment and microbial electrolysis greatly enhanced sludge hydrolysis and biohythane production. Illumina Miseq sequencing of 16S rRNA gene amplicons indicates that anode biofilm was dominated by exoelectrogenic *Geobacter*, fermentative bacteria and hydrogen-producing bacteria in the AS-MEC. The cathode biofilm was dominated by fermentative *Clostridium*. The dominant archaeal populations on the cathodes of AS-MEC and RS-MEC were affiliated with hydrogenotrophic *Methanobacterium* (98 %, relative abundance) and *Methanocorpusculum* (77 %), respectively. Multiple pathways of gas production were observed in the same MEC reactor, including fermentative and electrolytic H_2_ production, as well as hydrogenotrophic methanogenesis and electromethanogenesis. Real-time quantitative PCR analyses showed that higher amount of methanogens were enriched in AS-MEC than that in RS-MEC and RS-OCMEC, suggesting that alkali-pretreated sludge and MEC facilitated hydrogenotrophic methanogen enrichment.

**Conclusion:**

This study proves for the first time that biohythane could be produced directly in biocathode MECs using waste sludge. MEC and alkali-pretreatment accelerated enrichment of hydrogenotrophic methanogen and hydrolysis of waste sludge. The results indicate syntrophic interactions among fermentative bacteria, exoelectrogenic bacteria and methanogenic archaea in MECs are critical for highly efficient conversion of complex organics into biohythane, demonstrating that MECs can be more competitive than conventional anaerobic digestion for biohythane production using carbohydrate-deficient substrates. Biohythane production from waste sludge by MEC provides a promising new way for practical application of microbial electrochemical technology.

**Electronic supplementary material:**

The online version of this article (doi:10.1186/s13068-016-0579-x) contains supplementary material, which is available to authorized users.

## Background

Hythane is an emerging alternative fuel that contains a mixture of hydrogen and methane. By blending a small percentage of hydrogen (5–10 %) with methane in natural gas or biogas, studies showed that the combustion rate was enhanced and the lean limit of combustion was extended, which greatly increased the efficiency of methane-powered vehicles [1, 2]. Biohythane (biohydrogen and biomethane) is hythane produced from renewable biomass such as wastewater or solid waste, which gained great attention recently due to its great advantages of simultaneous waste treatment and energy production. Biohythane has been produced from different organic waste such as food wastes, agricultural residues and municipal solid wastes using two-phase anaerobic digestion [3–6], in which the integration of biohydrogen from dark fermentation and biomethane from methanogenesis showed a feasible approach for energy-neutral waste treatment.

Waste sludge disposal is among the most difficult tasks faced by wastewater treatment facilities. Anaerobic digestion (AD) is generally used to stabilize and reduce sludge volume and produce biogas [7, 8]. However, the quality of the biogas as a renewable fuel is not ideal, and the economic value of biogas is low. ADs in wastewater treatment facilities produce inconsiderable amount of hydrogen because of the low content of carbohydrates in sludge flocs and hydrogen consumption by methanogenesis. Hydrogen and methane have also been generated from different organic waste using microbial electrolysis cells (MECs), in which sludge has been used as inoculum or direct substrate [9–14]. MECs use exoelectrogenic microorganisms to break down organics and transfer electrons to an external circuit. If an external voltage (0.4–0.8 V) is applied to further reduce the cathode potential, hydrogen can be produced at a high yield [15–17]. Great progress has been made in MEC materials, architectures, and comprehension of microbial ecology [18], and the substrates used in MECs have evolved from simple organics to complex and actual waste such as winey wastewater, domestic wastewater, landfill leachate and waste sludge [19–25]. To date all MEC studies have focused on either biohydrogen production or biomethane production, but no group has reported biohythane production from MECs. In fact, methanogenesis has been considered as a major issue in hydrogen-producing MECs without effective solutions.

In this study, we report for the first time biohythane production from sludge with a higher production rate by MEC than that by anaerobic digestion. The production of biohythane from MECs not only improves hythane production from complex waste using a new technology, it also expands the niche application of MECs for waste treatment. Instead of focusing on the challenging pathways of pure gases (H_2_ or CH_4_), MECs can be more practical in producing a higher value biohythane as a mixed energy carrier. Furthermore, we replaced the expensive metal catalysts on the cathode with self-sustaining biocathode [26–28]. In biocathode MECs, electroactive microorganisms capable of receiving electrons from the cathode facilitate bioelectrosynthesis or electrofermentation [29, 30]. We analyzed the microbial community structure and interactions using Illumina Miseq sequencing and real-time quantitative PCR of 16S rRNA gene, and revealed that the different microbial functional populations engaged in multiple syntrophic relationships in the waste sludge fed MEC reactors [31–33].

## Results

### Biohythane production from waste sludge in biocathode MECs

**Fig. 1:**
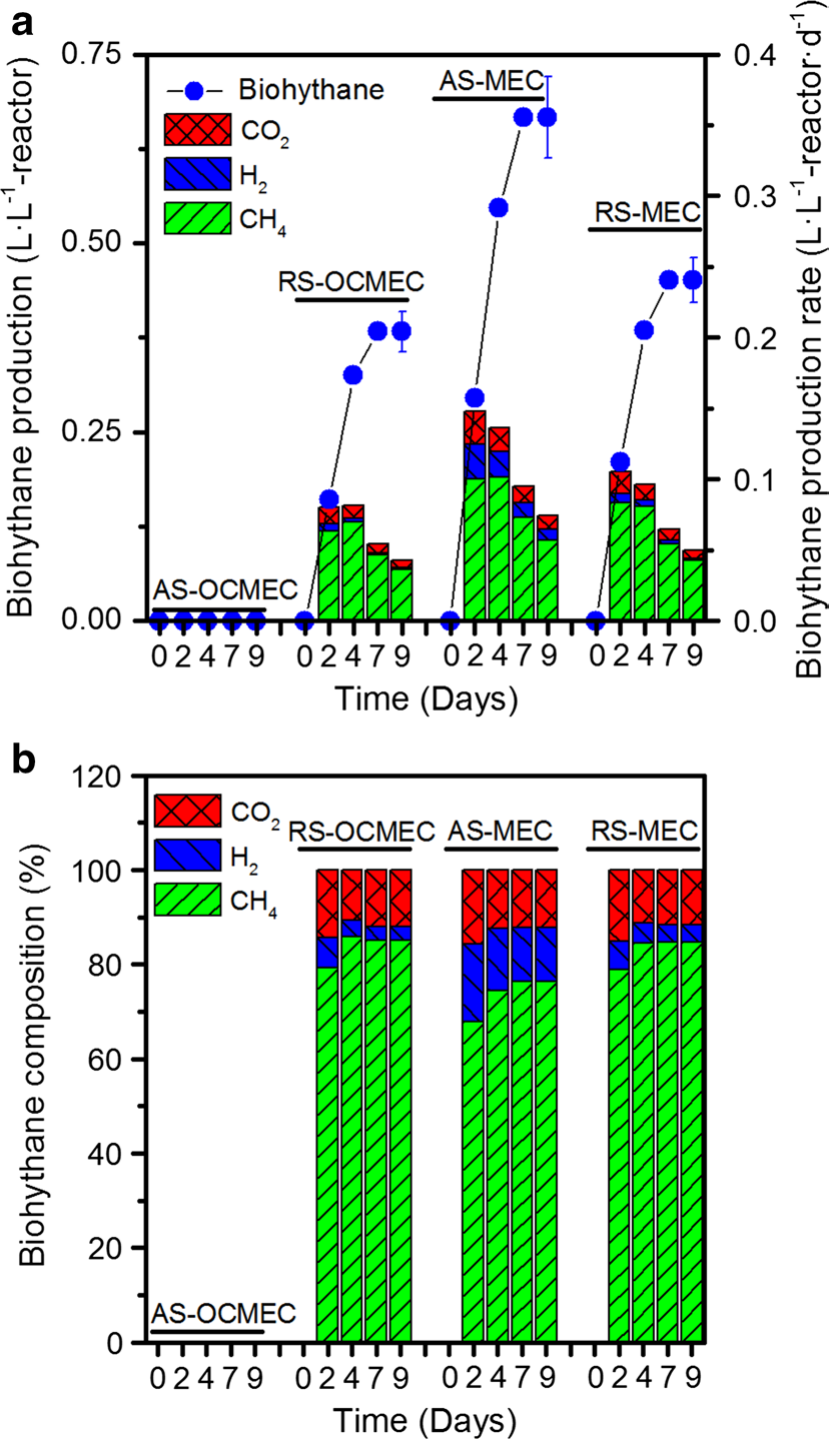
Biohythane production **(a)** and biohythane composition **(b)** in MECs during a steady operation cycle. The *circle lines* represent biohythane production (on the *left*), the last point with standard deviation were calculated based on three cycles of two duplicate reactors. The *columns* represent biohythane production rate (on the *right*). RS-OCMEC: raw sludge fed open-circuit MEC; AS-OCMEC: alkali-pretreated sludge fed open-circuit MEC; RS-MEC: raw sludge MEC with an applied voltage of 0.6 V; AS-MEC: alkali-pretreated sludge fed MEC with an applied voltage of 0.6 V

Biohythane production rate and gas composition of three fed-batches in different MEC reactors after 1 month of operation are presented in Fig. 1. During the 9 days of a fed-batch cycle, biohythane production of 0.667 ± 0.054 L·L^−1^-reactor (based on three fed-batch cycles) in alkali-pretreated sludge fed MECs (AS-MEC) was obtained, compared to 0.451 ± 0.030 L·L^−1^-reactor in raw sludge fed MEC (RS-MEC) and 0.383 ± 0.027 L·L^−1^-reactor in raw sludge fed open circuit MEC (RS-OCMEC) (Fig. 1a). The AS-MEC showed the highest biohythane production rate of 0.148 L·L^−1^-reactor·day^−1^ during the first 2 days, in which methane accounted for 67.8 %, with a production rate of 0.1 L·L^−1^-reactor·day^−1^, while hydrogen production rate was 0.025 L·L^−1^-reactor·day^−1^ and represented 16.7 % of the total gas. These rates were much higher than other reactors. The RS-MEC showed 0.083 L·L^−1^-reactor·day^−1^ of methane and 0.006 L·L^−1^-reactor·day^−1^ of hydrogen, while the RS-OCMEC produced 0.064 L·L^−1^-reactor·day^−1^ of methane and 0.005 L·L^−1^-reactor·day^−1^ of hydrogen) (Fig. 1a). No methane or hydrogen was detected in the alkali-pretreated sludge fed open circuit MECs (AS-OCMEC) for 35 days, presumably due to the lack of methanogens that could directly utilize substrates in the anaerobic digestion control.

In this experiment, almost 95 % hydrogen and 80–85 % methane were produced in the first 4 days among all biohythane-producing reactors. During a 9-days operation, the average hydrogen production rate of AS-MEC, RS-MEC and RS-OCMEC were 0.011, 0.0023 and 0.0016 L·L^−1^-reactor·day^−1^, respectively. The average percentage of hydrogen of three fed-batch cycles in AS-MEC, RS-MEC and RS-OCMEC, reached up to 11.3, 3.61 and 2.94 % (Fig. 1b), respectively, indicating the gas mixture in AS-MEC could be used as biohythane (5–15 % hydrogen addition). The current density versus time in the MEC fed with untreated and alkali-pretreated sludge was different at an applied voltage of 0.6 V (Additional file 1: Figure S1). The maximum current density of the AS-MEC (62 A/m^3^) was nearly two times higher than that of the RS-MEC (23 A/m^3^).

**Fig. 2:**
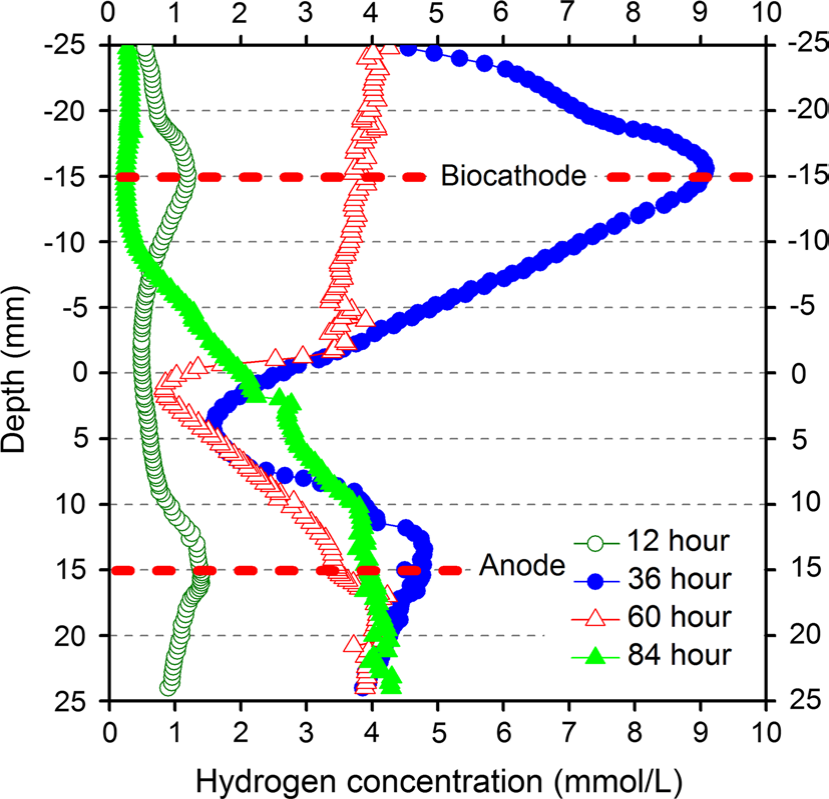
Hydrogen concentration in AS-MEC was measured in situ by H_2_ microsensor. The distance from the biocathode to the middle part of anode is about 30 mm

Hydrogen variations in the AS-MEC were measured in situ using a hydrogen microsensor (Fig. 2). Hydrogen concentration in the near-cathode region reached a maximum peak (9 mmol/L) in 36 h, and then decreased in 60 and 84 h, suggesting hydrogen produced on the cathode was consumed by hydrogenotrophic methanogens. However, hydrogen concentration in the near-anode region increased over time and then decreased slightly after 36 h. The hydrogen in the near-anode region was mainly attributed to anaerobic fermentation rather than hydrogen diffusion from the biocathode, because Fig. 2 showing a consistent hydrogen concentration gradient across the two electrodes, the lowest level of hydrogen was consistently found in the middle of the two electrodes, leading to a trough-shaped hydrogen profile.

### Variations of soluble organic matters

**Fig. 3:**
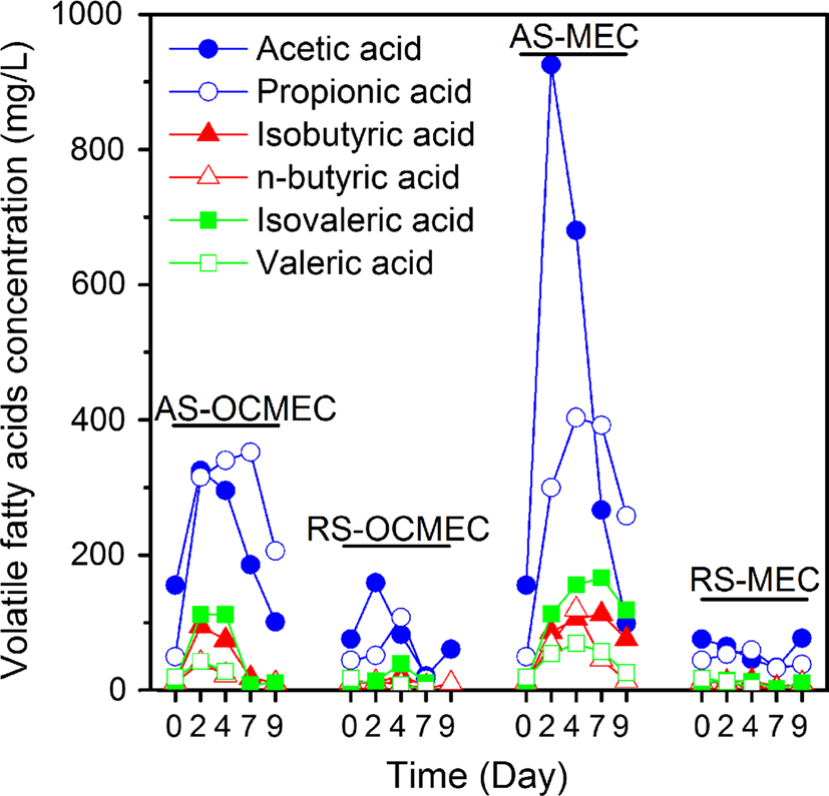
VFAs concentration in different MECs during a fed-batch cycle. RS-OCMEC: raw sludge fed open-circuit MEC; AS-OCMEC: alkali-pretreated sludge fed open-circuit MEC; RS-MEC: raw sludge fed MEC with an applied voltage of 0.6 V; AS-MEC: alkali-pretreated sludge fed MEC with an applied voltage of 0.6 V

Figure 3 shows the effects of pretreatment on sludge degradability and volatile fatty acids (VFA) concentration. The alkali-pretreatment increased the initial VFA concentration in the reactors (AS-OCMEC, AS-MEC) as compared with the raw sludge (RS-OCMEC, RS-MEC). More importantly, the alkali-pretreatment greatly increased the sludge degradability as evidenced by the dramatic increase in VFA concentration during the first 2 days of operation of AS-MEC from 260 to 1550 mg/L. Similar trend was observed in AS-OCMEC as well with a smaller increase from 260 to 930 mg/L (Fig. 3). Acetic acid was the main VFA product, which is favorable due to its easy conversion to current by exoelectrogens. The VFA concentration decreased sharply after day 4 due to microbial consumption, and higher current was produced during the same period of time (Additional file 1: Figure S1).

Alkaline pretreatment also greatly enhanced the release of soluble organics from waste sludge. Soluble protein concentration in the AS-MEC increased to 2300 mg/L or by 16-fold of raw waste sludge (Additional file 1: Figure S2). Meanwhile, carbohydrates were substantially elevated from 10 to 380 mg/L (Additional file 1: Figure S2). During the first 2 days, soluble protein and carbohydrates contents decreased substantially and VFAs concentration increased rapidly. After 4 days, the concentrations of protein and carbohydrates leveled off but soluble chemical oxygen demands (SCOD) took on slowly decline (Additional file 1: Figure S2). In contrast, the soluble organics in RS-MEC and open circuit MEC (OCMEC) changed much less than that in AS-MEC.

### Bacterial community structures of the biofilms in MECs

**Fig. 4:**
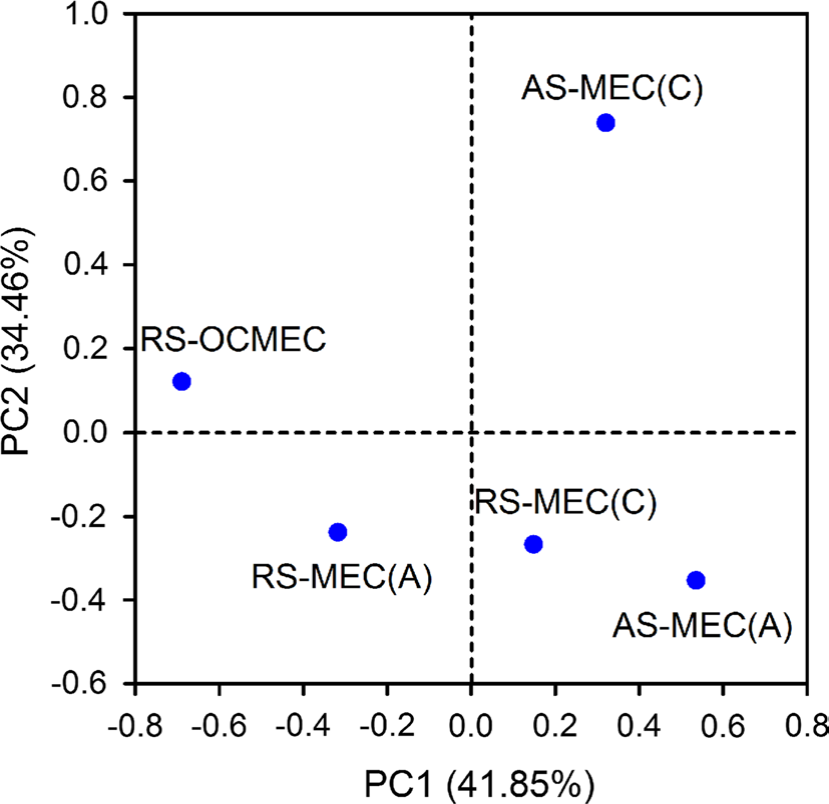
Principal component analysis (PCA) based on operational taxonomic units of different anode (A) and cathode (C) biofilms of MECs and anaerobic digested sludge of RS-OCMEC

Illumina Miseq sequencing showed that over 12,900 high-qualified 16S rRNA sequences with an average length of 395 bp for each sample were obtained (Additional file 1: Table S1). Total 492, 648, 617, 405 and 531  operational taxonomic units (OTUs) were determined at 97 % similarity for communities of RS-OCMEC, the anode and cathode of RS-MEC, and the anode and cathode of AS-MEC, respectively. The bacterial communities of biofilms in AS-MEC (fed with alkali-pretreated sludge) have relative lower diversity (Shannon indices of 3.64 and 3.81 for anode and cathode of AS-MEC) than that in RS-MEC (4.77 for anode and 4.33 for cathode of RS-MEC), which suggested alkali-pretreatment resulted in the extinction of some species. Principal component analysis (PCA) based on OTUs show that the different samples are separated from each other, indicating distinct microbial communities presented in different conditions (Fig. 4).

**Fig. 5:**
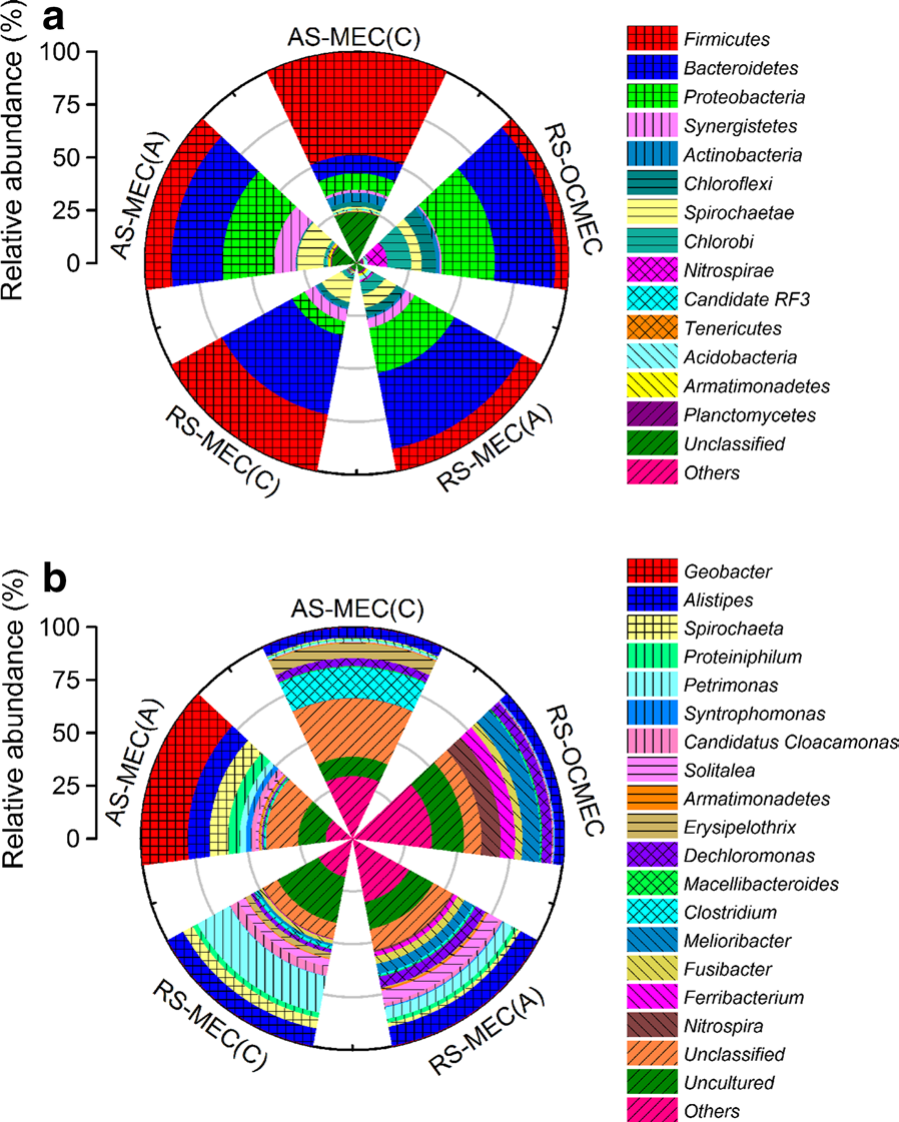
Microbial community taxonomic wind-rose plots based on relative abundance of 16S rRNA sequences of sludge and biofilms in MEC at the bacterial phylum (**a**) and genus levels (**b**)

*Bacteroidetes*, *Proteobacteria* and *Firmicutes* accounted for 59–71 % of the total sequences in each community at phylum level (Fig. 5a). The relative abundances of *Firmicutes* in the biocathode biofilms of RS-MEC and RS-MEC were 27 and 48 %, respectively, which were much higher than that in the anode biofilms of RS-MEC (10 %) and AS-MEC (12 %). The percentages of *Bacteroidetes* in the anode (37 %) and biocathode (38 %) biofilms of RS-MEC were higher than that in the anode (24 %) and biocathode biofilm (9 %) of AS-MEC. The relative abundances of *Proteobacteria* were 22–24 % in the anode biofilm of RS-MEC and AS-MEC, compared with 7–8 % in the biocathode biofilm in RS-MEC and AS-MEC.

The microbial community structures in the anode and cathode biofilms were obviously different in MECs (Fig. 5b). *Geobacter* (22 %) as a typical exoelectrogenic microbe was the majority of dominant populations in the anode biofilm of AS-MEC, followed by *Alistipes* (10 %), *Spirochaeta* (9 %), *Proteiniphilum* (6 %) and *Petrimonas* (3 %) (Fig. 5b). By contrast, the majority of predominant populations in the cathode biofilm of AS-MEC belonged to *Clostridium* (15 %). The predominant genera were affiliated with *Alistipes* (9 %), *Solitalea* (6 %), *Petrimonas* (5 %) and *Dechloromonas* (5 %) in the anode biofilm of RS-MEC, while the predominant populations belonged to *Spirochaeta* (5 %) and *Petrimonas* (17 %) in the biocathode biofilm.

### Archaeal community structures and quantity of the biofilms in MECs

**Fig. 6:**
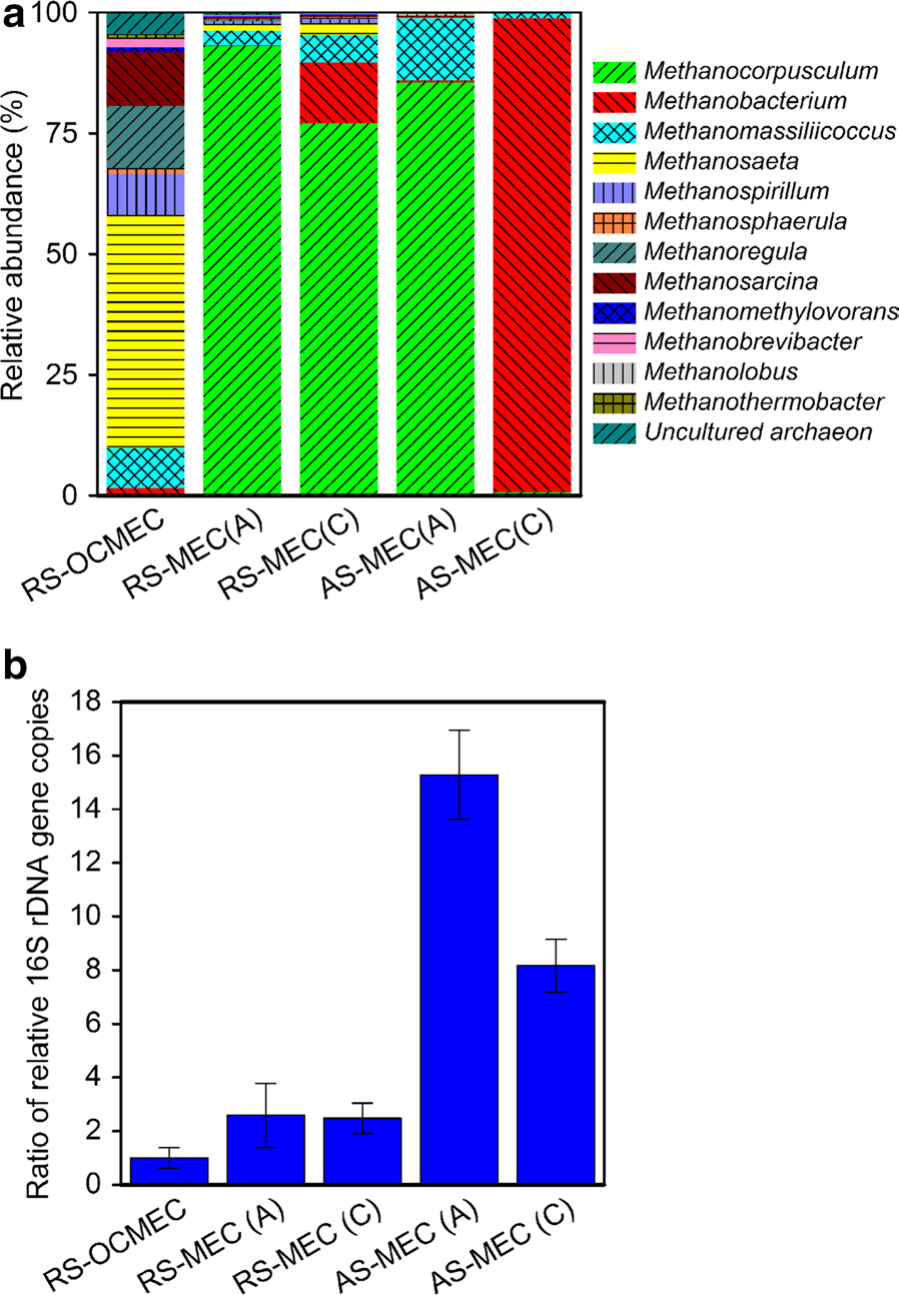
Microbial community taxonomic histogram based on relative abundance of 16S rRNA sequences of sludge and biofilms in MEC at the archaeal genus level (**a**) and relative quantification PCR of archaea with 16S rRNA gene copies (**b**)

High-throughput sequencing of 16S rRNA gene indicated that the majority of the predominant archaeal populations belonged to *Methanocorpusculum* (77–85 %) in the biofilms of the electrodes of RS-MEC and AS-MEC except AS-MEC biocathode where *Methanobacterium* (98 %) was dominant methanogen (Fig. 6a). By contrast, the most predominant genus in RS-OCMEC was affiliated with *Methanosaeta* (48.2 %). Archaeal 16S rRNA genes copies of the biocathode and anode biofilms in AS-MEC were 8 and 16 times as high as that in RS-OCMEC (Fig. 6b), while the 16S rRNA genes copies of RS-MEC (A) were similar to RS-MEC (C) and 2 times as high as that of RS-OCMEC. The results indicate that alkali-pretreatment and microbial electrochemical system facilitated methanogen enrichment. Methanogens were enriched effectively in the anode and biocathode biofilms of MECs.

## Discussion

### MECs enhance the hydrolysis of waste sludge and biohythane production

This study proves for the first time that biohythane could be produced directly in biocathode MECs using raw or alkali-pretreated waste sludge. MECs showed the highest biohythane production from alkali-pretreated sludge. Both MECs and conventional anaerobic digestion (open-circuit MEC) showed considerable biohythane production using raw sludge. No methane and hydrogen were detected in alkali-pretreated sludge fed open circuit MEC (AS-OCMEC) during a 9-d operation (Fig. 1). The community analyses indicated that archaeal community in RS-OCMEC dominated by an acetoclastic *Methanosaeta* [34], while the majority of dominant archaeal populations in MECs belonged to hydrogenotrophic methanogens (*Methanocorpusculum* and *Methanobacterium*) (Fig. 6a). Obviously, the alkali-pretreatment suppressed acetoclastic methanogens in the raw waste sludge and facilitated the acidogenesis that provide the VFAs for exoelectrogen enrichment. Nevertheless, hydrogenotrophic methanogens or electromethanogens prevailed fast and contributed to biohythane production in MECs during a 9-d operation, not in AS-OCMEC.

These results have showed that MEC has advantage of fast reaction velocity compared with anaerobic digestion as reported previously [10, 22, 25, 35]. The biogas component of AS-MECs was almost consistent with commercial hythane [2]. Alkali-pretreatment played an important role in accelerating succedent decomposition of waste sludge, which enhanced biohythane production in AS-MECs (Fig. 1). The results showed that waste sludge is an appropriate substrate for biohythane production by MECs. In contrast with two-phase anaerobic digestion, biohythane production by MEC became more competitive using carbohydrate-deficient substrates. A recent study showed that high concentration methane of 95 % was produced from waste activated sludge using MEC at ambient temperature [24]. To optimize biohythane composition, organic loading rate, sludge retention time, temperature, substrate variety, cathode potential and system integration should be investigated in the future. The biohythane of a full-scale MEC reactor can be collected continuously using a gas storage tank before use in the industrial applications. The component of biohythane can be adjusted (5–20 % of hydrogen) using a gas blending systems to meet the end-use devices such as household appliances and vehicles, which approach is same as the hythane production.

The acetic acid concentration in AS-MEC was two times higher than that in open-circuit AS-MEC by day 2 (Fig. 3), suggesting that microbial electrochemical system facilitated the acidification of alkaline pretreated waste sludge compared with conventional anaerobic digestion [10]. As alkaline pretreatment destroyed sludge flocs and accelerated organic matter’s hydrolysis, the acidogenesis in open-circuit AS-MEC was better than that in RS-MEC and RS-OCMEC [35]. However, no methane was detected in AS-OCMEC in 9 days, suggesting the majority of acetoclastic methanogens in the initial raw sludge were lysed certainly during the alkali-pretreatment. MEC also accelerated methanogen enrichment that resulted in a higher biohythane production rate. Propionic acid as a central intermediate often accumulated in the degradation of complex organic matters, especially in methanogenic environments. VFAs analyses showed that propionic acid accumulation (200–300 mg/L) present in close and open circuit AS-MEC after 9 days, suggesting that enriching propionate-oxidizing acetogenic bacteria in MECs may further enhance biohythane production from waste sludge.

### Biohythane provides a new perspective to view methanogenesis in hydrogen-producing MECs

Hydrogen re-consumption by hydrogenotrophic methanogens in MECs has been a major challenge for hydrogen-producing MECs [36, 37]. To achieve a high yield and high purity of H_2_ in MECs, several methods including methanogen inhibitors (e.g., bromoethanesulfonate, lumazine), short hydraulic retention time, intermittent exposure to air and low temperatures have been used to depress methanogenesis [16]. The methanogens could be significantly repressed at the relatively low temperatures [16, 37], suggesting that MEC should be operated at 15 °C considering both hydrogen production and methanogenesis inhibition. Hydrogenotrophic methanogens will prevail over time when hydrogen-producing MECs using waste sludge are operated above room temperature. Biohythane as mixture of biomethane and biohydrogen produced from organic waste could be directly used in internal combustion engines, which offered an alternative approach to solve troublesome methanogenesis in hydrogen-producing MECs.

### Multiple syntrophic interactions drive cascade utilization of waste sludge in MECs

Syntrophy is an essential intermediary step in the anaerobic metabolism, especially for the complete conversion of complex polymers such as polysaccharides, proteins, nucleic acids, and lipids to methane [38]. Metabolic crossfeeding is an important process that can broadly shape microbial communities. Illumina Miseq sequencing and principal component analyses indicate that microbial community structures greatly distinguished from each other in samples obtained from different reactors (Figs. 4, 5). Diverse trophic groups in MECs belonged to primary/secondary fermentative bacteria (proteolytic and saccharolytic bacteria, hydrogen-producing bacteria), acetogenic bacteria, exoelectrogenic bacteria and hydrogenotrophic methanogenic archaea according to the taxonomic identification [39]. The predominant populations in the anode biofilm of AS-MEC were affiliated with *Geobacter* (22 %), *Alistipes* (10 %), *Spirochaeta* (9 %), *Proteiniphilum* (6 %) and *Petrimonas* (3 %). The relative abundance of exoelectrogenic *Geobacter* was higher in AS-MEC than that in other MECs, which is consistent with the findings of higher current production because *Geobacter* is the most efficient exoelectrogen using acetate reported in literature. *Alistipes* can produce VFAs and hydrogen using protein and carbohydrates [40]. *Spirochaeta* as saccharolytic bacterium is responsible for decomposition of (poly) carbohydrates and production of acetate, carbon dioxide and hydrogen [41]. *Proteiniphilum* as proteolytic bacterium is capable of producing acetic and propionic acids using yeast extract, peptone and arginine [42], and its relative abundance increased with the order of RS-OCMEC, RS-MEC and AS-MEC. *Petrimonas*, an acidogenic bacterium, can degrade protein and carbohydrates, which was also reported in previous studies as a predominant genus in sludge fed MECs [11, 43]. The majority of predominant genera in the cathode biofilm of AS-MEC belonged to putative hydrogen-producing *Clostridium* (15 %). The sequencing analyses indicated putative fermentative hydrogen-producing bacteria were enriched in both electrode biofilms, and hydrogen production on the electrodes was also proved by hydrogen microsensor measurements (Fig. 2).

Archaeal community analyses indicated that the majority of methanogenic populations was affiliated with hydrogenotrophic *Methanocorpusculum* (relative abundance of 85 %) and *Methanobacterium* (98 %) in the anode and cathode biofilms of AS-MEC, respectively (Fig. 6a). *Methanobacterium* capable of electromethanogenesis has been reported, which was the most predominant methanogen in the cathode biofilm of electromethanogenic MEC using inorganic carbon source [29]. The predominant populations in the biofilms proved that hydrogen production by fermentation and electrolytic process, hydrogenotrophic methanogenesis and electromethanogenesis occurred simultaneously in the single-chamber MECs.

The microbial community structure reveals that different functional groups interacted synergistically in the MEC reactors to convert recalcitrant sludge into biohythane. The multiple levels of interactions in these syntrophic consortia include three groups. First metabolic crossfeeding occurred between fermentative and acetogenic bacteria and exoelectrogenic bacteria. Fermentative and acetogenic bacteria also partnered with methanogenic archaea. Real-time quantitative PCR results showed that the amount of methanogens was higher in AS-MEC than that in RS-MEC and RS-OCMEC (Fig. 6b), suggesting that alkali-pretreatment and MEC facilitated hydrogenotrophic methanogen enrichment in the anode and cathode biofilms as hydrogen production. Compared to the cathode biofilm of AS-MEC, the anode biofilm enriched large amount of methanogens (Fig. 6b), implying that third syntropic interaction may occur between methanogenic archaea and exoelectrogenic bacteria on the anode as reported previously [44]. However, putative interspecies electron transfer between *Methanocorpusculum* and *Geobacter* should be further proved based on co-culture test.

## Conclusion

This study proved that biohythane could be produced directly in biocathode MECs using waste sludge. The highest biohythane production rate of 0.148 L·L^−1^-reactor·day^−1^ was obtained in the alkali-pretreated sludge fed MECs (AS-MEC), which was 80 % higher than that in the anaerobic digestion. Real-time quantitative PCR and VFAs results demonstrated that MEC and alkali-pretreatment accelerated enrichment of hydrogenotrophic methanogen and hydrolysis of waste sludge that resulted in a higher biohythane production. The most predominant population on the anode of AS-MEC was affiliated to exoelectrogenic *Geobacter*, while biocathode was dominated by fermentative *Clostridium*. The majority of methanogenic archaea on the cathodes of AS-MEC belonged to hydrogenotrophic *Methanobacterium*. The community analyses implied that multiple syntrophic interactions between fermentative bacteria, exoelectrogenes and methanogenic archaea in MECs drive biohythane production from waste sludge. Compared to anaerobic digestion, biohythane production by MEC became more competitive using carbohydrate-deficient substrates, and provided a new approach for bioenergy production using waste sludge.

## Methods

### Waste sludge pretreatment

Waste sludge from a secondary clarifier of the Harbin Wenchang wastewater treatment plant (Harbin, China) was used as the sole substrate in the study. The alkali-pretreatment of the initial sludge (with pH of 6.8 ± 0.1) was performed using 4 mol/L NaOH at adjusted pH 12 [35]. The treated samples had a pH 9–10 and stored at 4 °C for MEC studies. Right before the experiments, the sludge was mixed with 100 mM PBS (KCl, 0.13 g/L; NH_4_Cl, 0.31 g/L; NaH_2_PO_4_∙2H_2_O, 5.54 g/L; Na_2_HPO_4_∙12H_2_O, 23.11 g/L) according to 1:1 of the volume for pH conditioning. The final pH in the raw sludge (RS) and alkaline pretreated sludge (AS) that mixed with PBS was 7.2 ± 0.2 and 7.8 ± 0.2.

### MECs construction and operation

Single-chamber membrane-less MECs were constructed as previously described [45]. Each reactor had a volume of 40 mL, and carbon cloth with no catalyst was used as the cathode while carbon fiber brush served as the anode. All reactors were divided into two groups based on circuit connection: open circuit MECs were fed with either raw sludge (RS-OCMEC) or alkali-pretreated sludge (AS-OCMEC) as control test of anaerobic digestion, second group were closed circuit MECs (at an applied voltage of 0.6 V) fed with raw sludge (RS-MEC) or alkali-pretreated sludge (AS-MEC). All reactors were sparged for 20 min with ultra high purity (UHP) nitrogen (99.999 %) before each fed-batch experiment. All experiments were operated at 30 °C. The voltage across a serially connected external resistance (10 Ω) in each closed circuit MEC was recorded using a data acquisition system (Keithley 2700, OH). The MECs were refilled with raw or alkali-pretreated sludge when the current density of MECs decreased to 10–15 A/m^3^. All MECs were operated in batch mode for 2 months. All tests were conducted in two duplicate reactors.

### Biogas composition measurements

Hydrogen gas, methane and carbon dioxide in gaseous phase of MECs were measured using a gas chromatograph (Agilent GC7890a, America). Hydrogen concentration was detected by Unisense microsensor system. Prior to measuring hydrogen concentration in situ, the MEC reactor with alkali-pretreated sludge was operated for at least 3 replicates after steady performance and was vertically rotated 90° to make cathode upward for microsensor (10 *μ*m in diameter, Unisense, Denmark) insertion. Before the measurement, the hydrogen microsensor was polarized at +800 mV to reach a stable output and then was calibrated using a gas mixture controller.

### Organic components analysis

Chemical oxygen demand (COD) of solution in MECs after a whole cycle was measured after three steady fed-batch cycles according to the standard methods of American Public Health Association [46]. VFAs were analyzed by gas chromatograph (GC4890, Agilent, America). Protein concentration was gauged by UV-6000 spectrophotometer (METASH, China) with Modified BCA Protein Assay kit (Sangon Biotech, China). The content of polysaccharides was detected by phenol-vitriol colorimetry method [47]. Samples for VFAs, soluble COD, soluble protein and soluble polysaccharides characterization were obtained by filtering with 0.45 *μ*m filter membrane.

### Illumina sequencing analysis and *quantitative* PCR detection

Genomic DNAs of the electrode biofilms and bulk solution samples in parallel MECs were extracted by PowerSoil DNA Isolation Kit (Mobio laboratories, CA) according to the manufacturer’s protocol. DNA concentration and purity were detected by NanoPhotometer P-Class (Implen). Prior to PCR amplification, DNA from two parallel reactors were mixed. The V4-V5 region (length of ~400 bp) of bacterial and archaeal 16S rRNA gene was amplified separately using a set of primers: 515F (5′-GTGCCAGCMGCCGCGGTAA-3′) and 907R (5′-CCGTCAATTCCTTTR AGTTT-3′) for bacteria, 519F (5′-CAGCMGCCGCGGTAATWC-3′) and 915R (5′-GTGCTCCCCCGCCAATTCCT-3′) for archaea. After integrated with barcode, PCR amplification was implemented using ABI GeneAmp^®^ 9700 PCR system. High-throughput sequencing was performed on Illumina Miseq platforms according to the standard protocols. Raw sequencing data were filtered and analyzed using the pipelines of Quantitative Insights Into Microbial Ecology (QIIME) software (http://www.microbio.me/qiime). Operational taxonomic units (OTUs) were determined based on the threshold of 97 % similarity using UPARSE software (http://drive5.com/uparse/). Species diversity was evaluated in the MOTHUR (http://www.mothur.org). A representative sequence of each OTU was aligned for taxonomic identification using the Silva database (http://www.arb-silva.de) and Ribosomal Database Project (RDP) classifier (version 2.2 http://sourceforge.net/projects/rdp-classifier/) with a minimum confidence of 70 % [48, 49].

The DNA samples extracted from anaerobic digestion raw waste sludge (RS-OCMEC), anode and cathode biofilms of MEC without alkali-pretreatment [RS-MEC (A), RS-MEC (C)] and with alkali-pretreatment [AS-MEC (A), AS-MEC (C)] were used to quantify archaea copies. Archaeal universal primers 787F (5′-ATTAGATACCCSBGTAGTCC-3′) and 1059R (5′-GCCATGCACCWCCTCT-3′) were chose to amplify archaeal community [50]. The *q*-PCR reaction mixtures (25 μL) contained 1× SYBR Green *q*PCR Mix (Tiangen, China), 300 nM of each primer and 1 μL of template DNA. Amplifications were performed on an ABI 7500 Real-Time PCR System (Applied Biosystems). The protocol of PCR amplification consisted of two steps: initial denaturation for 2 min at 95 °C followed by 40 cycles of denaturation for 10 s at 95 °C, annealing for 15 s at 60 °C, elongation for 30 s at 68 °C. Standard curve was obtained using diluted DNA of RS-OCMEC sample and the efficiency value calculated was up to 1.06 with an R^2^ of 0.99. All relative *q*-PCR reactions were performed in triplicate.

## Additional file

10.1186/s13068-016-0579-x Similarity-based OTUs and species richness and diversity estimates of bacteria in different systems. **Figure. S1.** Current density of MEC fed with raw sludge (RS-MEC) and alkali-pretreated waste sludge (AS-MEC). **Figure. S2.** Variations of SCOD (A), soluble protein (B) and carbohydrates concentration (C) of raw sludge open-circuit MEC (RS-OCMEC), MEC fed with raw sludge (RS-MEC) or alkali-pretreated sludge (AS-MEC).

## Abbreviations

MECs: microbial electrolysis cells
COD: chemical oxygen demand
AS-MEC: alkali-pretreated sludge fed MEC
RS-MEC: raw sludge fed MEC
RS-OCMEC: raw sludge fed open circuit MEC
AS-OCMEC: alkali-pretreated sludge fed open circuit MEC
RS: raw sludge
AS: alkali-pretreated sludge
VFAs: volatile fatty acids
OTUs: operational taxonomic units
q-PCR: quantitative PCR
PCR: polymerase chain reaction
AD: anaerobic digestion
PCA: principal component analysis
