# Supplementary material for: Multiple syntrophic interactions drive biohythane production from waste sludge in microbial electrolysis cells
Source: Biotechnol Biofuels. 2016 Aug 2;9:162. doi: 10.1186/s13068-016-0579-x (PMC4971668; doi:10.1186/s13068-016-0579-x)
Supplement: Supplementary file 1 — 10.1186/s13068-016-0579-x Similarity-based OTUs and species richness and diversity estimates of bacteria in different systems. Figure. S1. Current density of MEC fed with raw sludge (RS-MEC) and alkali-pretreated waste sludge (AS-MEC). Figure. S2. Variations of SCOD (A), soluble protein (B) and carbohydrates concentration (C) of raw sludge open-circuit MEC (RS-OCMEC), MEC fed with raw sludge (RS-MEC) or alkali-pretreated sludge (AS-MEC). [file 13068_2016_579_MOESM1_ESM.pdf]

Page: 2; Table: 1; Figure: 2

Supplementary Information

The following information is provided to the article in *Biotechnology for Biofuels*

on

"Multiple syntrophic interactions drive biohythane production from waste sludge in biocathode microbial electrolysis cells"

Qian Liu[a], Zhiyong Jason Ren[b], Cong Huang[a], Bingfeng Liu[a], Nanqi Ren[a], Defeng Xing[a]*

[a] State Key Laboratory of Urban Water Resource and Environment, School of Municipal and Environmental Engineering, Harbin Institute of Technology, Harbin 150090, China

[b] Department of Civil, Environmental, and Architectural Engineering, University of Colorado Boulder, Boulder, CO 80309, USA

[*] **Corresponding author**. School of Municipal and Environmental Engineering, Harbin Institute of Technology, P.O. Box 2650, 73 Huanghe Road, Nangang District, Harbin, Heilongjiang Province 150090, China

E-mail address: dxing@hit.edu.cn

Tel& Fax: (+86) 451 86282195

Table. S1 Similarity-based OTUs and species richness and diversity estimates of bacteria in different systems.

Sample ID	Reads	0.97				
		**OTU**	**Ace**	**Chao**	**Shannon**	**Simpson**
RS-OCMEC	12985	492	533	530	4.6	0.0265
RS-MEC (A)	15189	648	744	757	4.77	0.0231
RS-MEC (C)	15836	617	736	740	4.33	0.0372
AS-MEC (A)	16736	405	491	498	3.64	0.0788
AS-MEC (C)	19932	531	615	628	3.81	0.0851

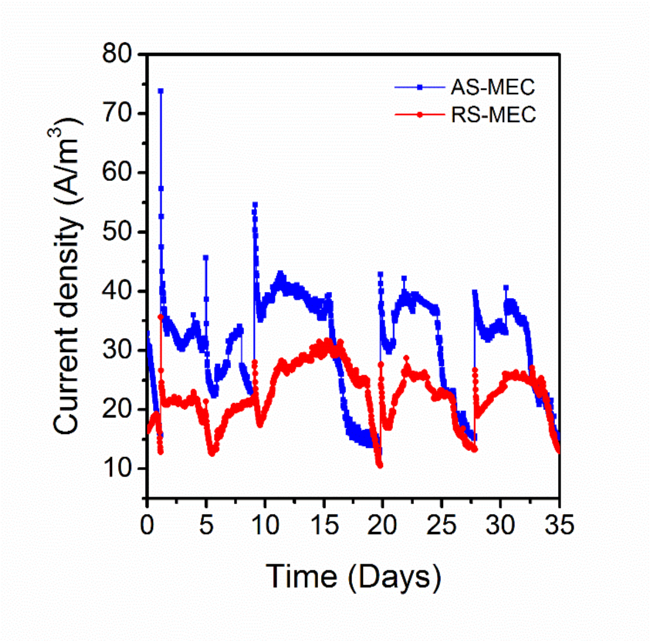

Fig. S1 Current density of raw sludge fed MEC (RS-MEC) and alkali-pretreated sludge fed MEC (AS-MEC).

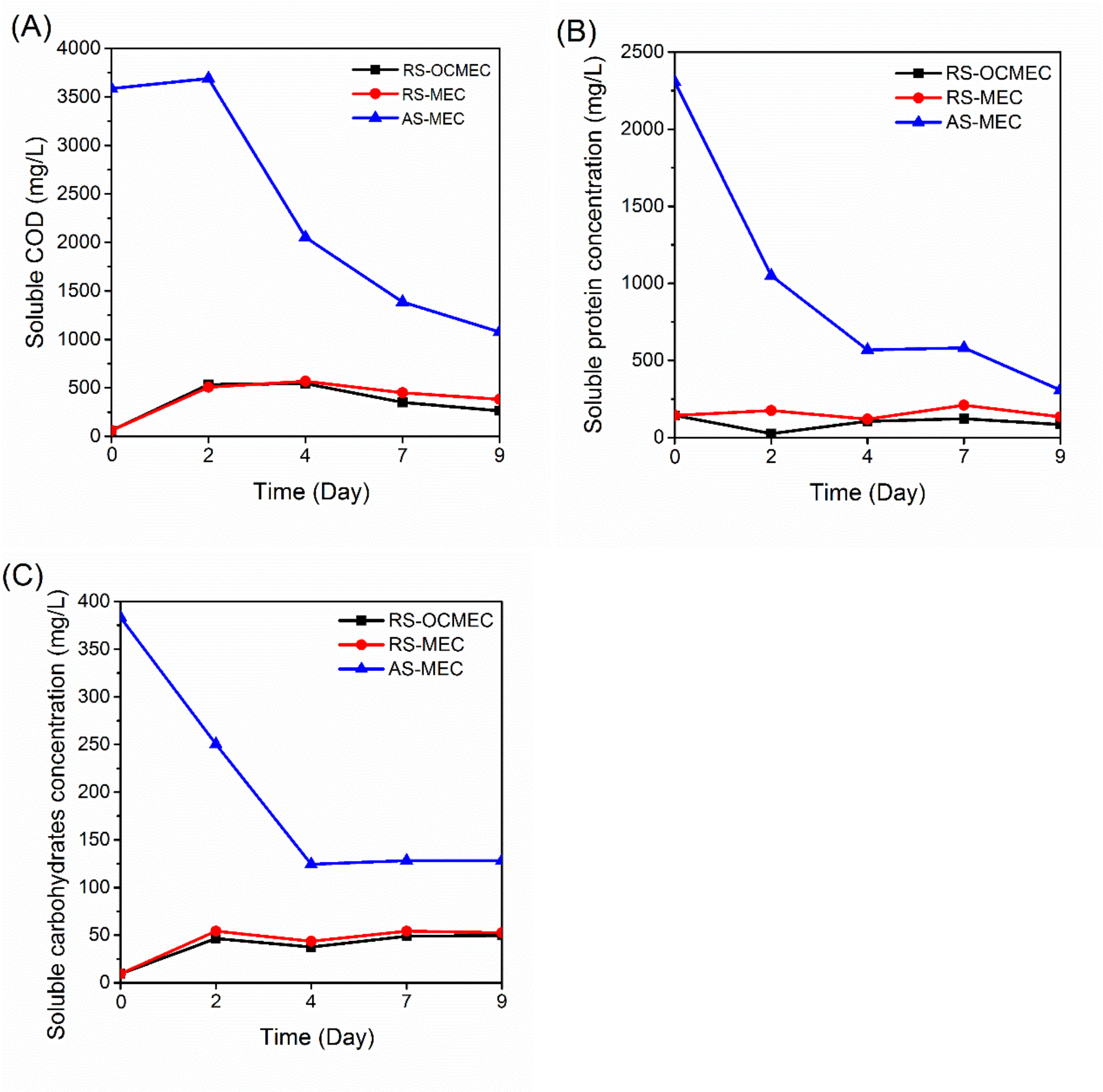

Fig. S2 Variations of SCOD (A), soluble protein (B) and carbohydrates concentration (C) of raw sludge fed open circuit MEC (RS-OCMEC), raw sludge fed MEC (RS-MEC) and alkali-pretreated sludge fed MEC (AS-MEC).
